# Systems approach to the study of brain damage in the very preterm newborn

**DOI:** 10.3389/fnsys.2015.00058

**Published:** 2015-04-14

**Authors:** Alan Leviton, Pierre Gressens, Olaf Wolkenhauer, Olaf Dammann

**Affiliations:** ^1^Neuroepidemiology Unit, Boston Children’s HospitalBoston, MA, USA; ^2^Department of Neurology, Harvard Medical SchoolBoston, MA, USA; ^3^Inserm, U1141Paris, France; ^4^Department of Perinatal Imaging and Health, Department of Division of Imaging Sciences and Biomedical Engineering, King’s College London, King’s Health Partners, St. Thomas’ HospitalLondon, UK; ^5^Department of Systems Biology and Bioinformatics, University of RostockRostock, Germany; ^6^Stellenbosch Institute for Advanced Study (STIAS)Stellenbosch, South Africa; ^7^Department of Public Health and Community Medicine, Tufts University School of MedicineBoston, MA, USA; ^8^Perinatal Epidemiology Unit, Department of Gynecology and Obstetrics, Hannover Medical SchoolHannover, Germany

**Keywords:** nuclear factor-kappa B (NF-κB), Notch-1, nuclear factor erythroid 2 related factor 2 (NRF2), inflammation, prematurity, brain, epigenetic, systems biology

## Abstract

**Background**: A systems approach to the study of brain damage in very preterm newborns has been lacking.

**Methods**: In this perspective piece, we offer encephalopathy of prematurity as an example of the complexity and interrelatedness of brain-damaging molecular processes that can be initiated inflammatory phenomena.

**Results**: Using three transcription factors, nuclear factor-kappa B (NF-κB), Notch-1, and nuclear factor erythroid 2 related factor 2 (NRF2), we show the inter-connectedness of signaling pathways activated by some antecedents of encephalopathy of prematurity.

**Conclusions**: We hope that as biomarkers of exposures and processes leading to brain damage in the most immature newborns become more readily available, those who apply a systems approach to the study of neuroscience can be persuaded to study the pathogenesis of brain disorders in the very preterm newborn.

## Introduction

Very preterm newborns are vulnerable to forms of brain damage that differ from those that occur in infants born near term (Volpe, 2011). Subsumed under the name of “encephalopathy of prematurity,” they include injuries/dysfunctions of cerebral white matter, as well as neuronal/axonal/synaptic disturbances. Increasingly, inflammation is recognized as a contributor to or correlate of the processes leading to these forms of damage (Adén et al., 2010; Van Steenwinckel et al., 2014), especially if prolonged or recurrent (Dammann and Leviton, 2014). We prepared this perspective piece to bring to the attention of the wider readership that this is potentially fruitful area of study by systems approaches.

This essay is divided into three parts. The first summarizes what is currently known about the antecedents and characteristics associated with brain damage in the very low gestational age newborn (ELGAN). The second part exemplifies the systems approach by describing the connectedness among pathways involved in inflammation ascendance and resolution, and among pathways involved with brain damage promotion and repair. Sometimes, the pathways and their components have pleiotropic properties, resulting in diverse effects including not only inflammation and inflammation resolution, but also brain damage as well as brain repair. The final part is our hopeful view of the future.

## Part 1: Why the Immature Brain is Vulnerable

We begin our illustration of systems epidemiology with the antecedents of perinatal brain damage in the very preterm newborn and with characteristics of the mother, placenta, and newborn that influence brain damage risk. The very preterm newborn is thought to be at heightened risk because of the combination of endogenous vulnerability and exposures.

Our linking endogenous vulnerability and these exposures to molecular pathways and networks achieves four goals. First, the exercise hints at the complexity of the processes involved. Second it identifies candidates for modulating the destructive processes, enhancing inflammation resolution and promoting brain repair needed to restore normal brain structure. Third, it brings systems epidemiology to the attention of those not familiar with it. Fourth, it points in a direction we hope others will consider traveling.

### Endogenous Vulnerability

By and large, the lower the gestational age, the higher the risk of brain damage (Locatelli et al., 2005; Himpens et al., 2008; Glinianaia et al., 2011; Sannia et al., 2013). Although low gestational age appears to be the best overall indicator of immaturity, not all infants born at 25 weeks of gestation are identically immature. Early indicators of physiologic instability, as identified with the Score for Neonatal Acute Physiology (SNAP) might provide supplemental information (and therefore more precision) about immaturity (Dammann et al., 2010).

The infant born too soon is not yet able to provide growth factors needed for normal growth (Sanders and Harvey, 2008), let alone for protection against perturbations (Dammann and Leviton, 1999). The influence of systemic physiologic instability remains uncertain (Bakewell-Sachs et al., 2009). The very preterm infant is born during the time her brain undergoes many developmental processes include those involved in laying the ground work for normal myelination, as well as those involved in the migration of subplate neurons from the germinal matrix to the thalamus and cortex, and synaptogenesis. These brain developmental processes in full swing at very low gestational ages appear to be especially vulnerable to disturbances (Back et al., 2007; Leviton and Gressens, 2007; McCarran and Goldberg, 2007; Verney et al., 2012).

The very preterm newborn seems more likely than the term newborn to exhibit intermittent or sustained systemic inflammation (ISSI; Dammann and Leviton, 2014). Such systemic inflammation in the very preterm newborn has repeatedly been an antecedent of brain damage (Nelson et al., 1998; Hansen-Pupp et al., 2008; Leviton et al., 2011a,b; O’Shea et al., 2012).

The preterm newborn appears to have limited ability to synthesize proteins with anti-inflammatory characteristics (Chheda et al., 1996; Jones et al., 1996; Blahnik et al., 2001). So does the immature rat brain (Brochu et al., 2011). In addition, components of the fetal systemic inflammatory response appear to be considerably more vigorous in very preterm newborns than in gestationally-older newborns (Rebuck et al., 1995; Berner et al., 1998; Nanthakumar et al., 2000; Rozycki et al., 2002; Schultz et al., 2002; Yoon et al., 2003; Athayde et al., 2005; Tatad et al., 2008). The result is that preterm newborns tend to have a pro-inflammatory imbalance in both the blood and brain.

An extremely low birth weight for gestational age appears to be a very good indicator of intra-uterine stimuli that promote epigenetic phenomena (Grissom and Reyes, 2013; Sookoian et al., 2013). This leads to the situation where an apparent indicator of endogenous vulnerability is really a reflection of processes that led to the intra-uterine growth restriction and the consequences of those processes. For example, growth-restricted newborns are more likely than others to be exposed post-natally to barotrauma, (Bose et al., 2009) which can lead to systemic inflammation (Bose et al., 2013). Whether because of epigenetic phenomena or other processes, severely growth-restricted newborns display a heightened inflammatory response, only part of which is explained by an increased need for assisted ventilation (McElrath et al., 2013). One possible explanation for this might be that pre-eclampsia, a known antecedent of intra-uterine growth restriction, contributes additionally to the adversities of very preterm newborn (Morsing and Maršál, 2014) but this remains controversial (Love et al., 2012). Another possible explanation is that hyperoxia-induced lung injury in animals involves inflammatory phenomena, (Weichelt et al., 2013; Martin et al., 2014), just as some of hyperoxia-induced cerebral white matter damage appears to be a consequence of inflammation, (Nold et al., 2013; Pham et al., 2014; Schmitz et al., 2014) or exacerbated by inflammation (Brehmer et al., 2012).

Some children born to obese women tend to score lower on measures of intelligence than their peers born to women who are neither overweight nor obese (Neggers et al., 2003; Helderman et al., 2012; Basatemur et al., 2013; Tanda et al., 2013). More studies have shown what appears to be a maternal obesity effect, than have failed to do so (Van Lieshout et al., 2011; Brion, 2013; Van Lieshout, 2013). Among the explanations invoked to explain the obesity-impaired development link are the neonatal inflammation associated with mother’s obesity (van der Burg et al., 2013), and epigenetic phenomena (Liu et al., 2014).

One example of a two-hit model begins with a sub-injurious noxious dose, which is followed by a fully-injurious dose. If these occur within a narrow time range, then the injury is greater than would occur without the earlier stimulus, resulting in what the authors identify as sensitization (Eklind et al., 2005; Mallard, 2012). Yet, with different time spans, the first of the two hits can appear to be protective (Mallard, 2012). This reduced probability and extent of damage in this case is called preconditioning (Hagberg et al., 2004) or tolerance (Lin et al., 2010).

### Exogenous Vulnerability

Many of the exposures associated with increased risk of brain damage are linked to systemic inflammation. We classify exposures by the time of their onset, distinguishing mainly between antenatal and postnatal exposures.

#### *In Utero* Exposures

Maternal characteristics, including pre-pregnancy body mass index, (Van Lieshout et al., 2011; Love et al., 2012; Basatemur et al., 2013; Casas et al., 2013; Hinkle et al., 2013; Kerstjens et al., 2013; Tanda et al., 2013), pregnancy weight gain, (Huang et al., 2014), and diet (Ojha et al., 2015), or their surrogates influence the child’s neurodevelopmental function. In addition, microbial invasion of the amniotic cavity (Romero et al., 2007; Fichorova et al., 2011) and histologic inflammation of the placenta and umbilical vessels (Hecht et al., 2011) have been associated with increased concentrations of inflammatory proteins in cord blood or blood obtained shortly after birth. Genito-urinary infections have also been associated with systemic inflammation in the newborn (Fichorova et al., 2015).

#### Early Postnatal Exposures

##### Bacteremia

Preterm newborns also have fragile skin, limited synthesis of complement components, antimicrobial proteins and peptides, and T(H)17-polarizing cytokine production, and multiple indwelling tubes, catheters, and lines, and all of which likely contribute to susceptibility to infection (Adkins, 2013; Cuenca et al., 2013). The frequently-occurring consequence of bacteremia can readily be followed/accompanied by systemic inflammation (Leviton et al., 2012).

##### Necrotizing enterocolitis and isolated intestinal perforation

Ligation of receptors of the innate immune system, including Toll-like receptors and the intracellular pathogen recognition receptor NOD2/CARD15 appear to be involved in the initiation of enterocyte apoptosis, which can destroy intestinal barriers and lead to necrotizing enterocolitis (Siggers and Hackam, 2011). Without destroying intestinal barriers, ligation of toll-like receptors might also lead to the inflammation that characterizes necrotizing enterocolitis (Afrazi et al., 2014; Lu et al., 2014). Thus, it is possible that necrotizing enterocolitis is a marker/consequence of inflammation, although it is probably more likely that necrotizing enterocolitis contributes to systemic inflammation (Martin et al., 2013).

##### Ventilation

Mechanical (pressure-limited/targeted) ventilation leads to systemic inflammation in very preterm newborns (Capoluongo et al., 2005; Turunen et al., 2006, 2011; Sarafidis et al., 2011; Bose et al., 2013). Although replacing pressure-limited ventilation equipment with volume-targeted equipment has the potential to reduce ventilation-induced lung injury and inflammation and their consequences (Peng et al., 2014), the avoidance of invasive ventilation is increasingly recognized as perhaps the optimal strategy (Strueby and Thébaud, 2014).

One explanation for the systemic inflammation accompanying lung injury invokes correlates of autophagy, a lysosomal degradation pathway that can eliminate (usually damaged) cytoplasmic components, including intracellular remnants of invasive microorganisms (Mizumura et al., 2012). Autophagy can also be induced by pro-inflammatory inducers or promoters (Levine et al., 2011), and inhibited by proteins that have anti-inflammatory characteristics (IL-4, IL-5, IL-6 and IL-10). Compared to their peers, mice with impaired autophagy have less lung injury after ventilation, as well as much less activation of the canonical nuclear factor kappa-light-chain-enhancer of activated B cells (NF-κB) pathway (López-Alonso et al., 2013). Here we have an example of how understanding of molecular processes appears to hold the promise of explaining what might account for what epidemiologists report. On the other hand, what applies to the lung might not apply to the brain (François et al., 2014).

##### Nutrition

Optimizing protein and energy intake and balance in the neonatal period primarily influences cognition, while relative energy deficiency appears to result in smaller total brain volumes (Keunen et al., 2015). Nevertheless “meta-analyzes of trial data have not provided convincing evidence for supplementation with specific nutrients to improve developmental outcomes.

##### Low socio-economic state

Low socio-economic state might contribute to diminished/ impaired function in ELGANs through multiple means and times along the course of development (Miller et al., 2009; Lucassen et al., 2013; Surén et al., 2013). Among very preterm newborns, those born to women who have characteristics of low social class tend to do less well than their peers born to women of higher socioeconomic status (Wang et al., 2008; Huhtala et al., 2012; Morinis et al., 2013). Indeed, regardless of gestational age at the time of birth, children reared in settings characterized by insecurity, stress, and diminished stimulation tend to do less well than their peers raised in more secure and stimulating environments (Bradley and Corwyn, 2002). Among the measurable effects are smaller brain volumes (Hanson et al., 2013) and lower scores on measures of overall language as well as receptive and expressive language skills (Wild et al., 2013).

Some of the limitations of children reared in disadvantaged homes have been attributed to the plethora of diminished opportunities for learning (Evans and Kim, 2010). More recently, epigenetic phenomena have been invoked as explanations for the association between social disadvantage early in life and later dysfunction (Ehlert, 2013; Tung and Gilad, 2013; Saban et al., 2014).

## Part 2. Molecular Consequences of Exposure to Inflammatory Stimuli

This section should be viewed as our attempt to show the interrelatedness among diverse pathways linking inflammation with brain damage in the very preterm newborn. We focus on three pathways nuclear factor-kappaB (NF-κB), Notch, and nuclear factor erythroid 2 related factor 2 (NRF2), whose connectedness will illustrate some of the interrelationships/complexities we consider most important. Each influences the other two. Each is closely linked to inflammation, and each has nervous system effects that go beyond inflammation.

Innate immune mechanisms include the inflammatory reactions of neutrophils and monocytes, usually triggered by organisms, their components or products. An inflammatory stimulus influences the expression of thousands genes (Zak and Aderem, 2009; Orozco et al., 2012). Perhaps that is why after severe trauma and burn injury, the circulating leukocyte transcriptome provides evidence of a “genomic storm” (Xiao et al., 2011).

The inflammatory response is characterized by a set of complex, cascading non-linear processes mediated by a large array of immune cells and inflammatory cytokines (Rankin, 2004).

Coordination of this complex response is achieved with transcription factors, which are proteins that bind to DNA and regulate gene expression (Medzhitov and Horng, 2009). Operating at multiple levels and differently in different tissues, they influence each cell’s sensitivity to inflammatory stimuli and response capabilities, as well as regulating signaling pathways and gene expression. Roughly 8% of genes in the human genome encode transcription factors.

The innate immune system is activated when receptors on local macrophages, such as Toll-like receptors (TLRs), recognize pathogen-associated molecular patterns (PAMPs), usually bacterial or viral components such as lipopolysaccharide, or endogenous damage-associated molecular patterns (DAMPs) (including those released by injured cells) (Kong and Le, 2011; Lin et al., 2011). Toll-like receptors also recognize thrombin (Babu et al., 2012) as well as other extravascular blood components (Wang, 2010), perhaps explaining why the occurrence of blood components in the extravascular space in developing white matter might contribute to local damage (Adler et al., 2010).

The ligation of Toll-like receptors (TLRs) activates NF-κB, AP1, CCAAT/enhancer binding protein delta (CREB), c/EBP, and IRF transcription factors (Newton and Dixit, 2012), as well as the mitogen-activated protein kinase (MAPK) pathway (Arthur and Ley, 2013). The response to this activation includes the production and release of inflammatory mediators, such as cytokines and chemokines. These, in turn, activate endothelial cells of local blood vessels resulting in the synthesis and release of adhesion molecules and the ability to recruit circulating leukocytes to the area, and allow them access to the inflamed tissue. Newly-arrived leukocytes are activated by cytokines released by the local inflammatory cells and thereby become able to eliminate the invaders or damaged tissue.

Transcription factors often function together to regulate components of the inflammatory response. For example, three transcription factors, NF-κB1 (an initiator), C/EBPdelta (an amplifier) and ATF3 (an attenuator) form a regulatory circuit that discriminates between transient and persistent Toll-like receptor 4-induced signals (Litvak et al., 2009).

An overly simplistic view of these three pathways is that the anti-inflammatory properties of NRF2 have the potential to modulate the pro-inflammatory tendencies of NF-κB. All three pathways can be viewed as pleiotropic. Nevertheless the authors of one paper felt compelled to write, “One of the greatest challenges in studying Notch signaling is the inability to predict the outcome of Notch activation, owing to its multiple roles” (Ables et al., 2011), while others wrote, “Although, this pathway is remarkably short, with no second messenger involved, it regulates expression of more than hundred target genes in a tissue-specific manner” (Borggrefe and Liefke, 2012). In addition to the contribution of context and cross-talk, some of the multiplicity and diversity of the pleiotropic functions of transcription factors have been attributed to epigenetic tendencies (Sarnico et al., 2012).

**Figure 1:**
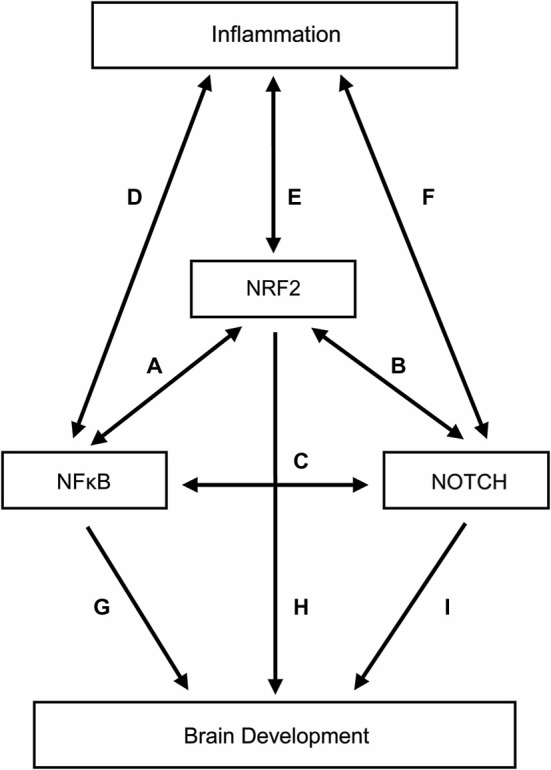
**Relationships among inflammation, three transcription factors (NF-κB, NRF-2, and Notch), and brain development**. To minimize complexity, the contributions of NF-κB, NRF-2, and Notch to brain damage in adults are not included in this figure. The letters adjacent to the lines identify a selected set of citations that support the existence of a relationship indicated by the line. **(A)** (Liu et al., 2008; Buelna-Chontal and Zazueta, 2013; Ganesh Yerra et al., 2013; Sandberg et al., 2013; Yang et al., 2013a,b; Djordjevic et al., 2015). **(B)** (Yang et al., 2013a; Kim et al., 2014; Wakabayashi et al., 2014). **(C)** (Ang and Tergaonkar, 2007; Hurlbut et al., 2007; Scholzke and Schwaninger, 2007; Osipo et al., 2008; Poellinger and Lendahl, 2008; Yang et al., 2013a; Yao et al., 2013b; Li et al., 2014; Yu et al., 2014). **(D)** (Covert et al., 2005; Werner et al., 2005; Liu et al., 2008; O’Dea and Hoffmann, 2009; Kim et al., 2011; Ruland, 2011; Bartuzi et al., 2013; Bakunina et al., 2015; Zhao et al., 2015). **(E)** (Kumar et al., 2012; Brune et al., 2013; Ruiz et al., 2013; Sandberg et al., 2013; Bakunina et al., 2015; Zhao et al., 2015). **(F)** (Hoyne et al., 2001; Arumugam et al., 2006; Fung et al., 2007; Fernandez et al., 2008; Palaga et al., 2008; Ito et al., 2012; Wongchana and Palaga, 2012; Zhang et al., 2012; Radtke et al., 2013; Yao et al., 2013b; Cheng et al., 2015). **(G)** (O’Dea and Hoffmann, 2009; Crampton and O’keeffe, 2013; Snow et al., 2014). **(H)** (Gupta et al., 2013). **(I)** (Wang et al., 1998; Oya et al., 2009; Imayoshi et al., 2013; Yao et al., 2013a; Kotasová et al., 2014; Schwanbeck, 2015).

We provide just a hint of the complexity of these three pathways and their interconnections in the Figure 1. Not shown in the Figure 1 are the relationships between adult brain diseases and NF-κB (Cai, 2009; Ridder and Schwaninger, 2009; Harari and Liao, 2010; Nogueira et al., 2011; Nestler, 2012; Sako et al., 2012; Crampton and O’keeffe, 2013; Hoesel and Schmid, 2013; Mc Guire et al., 2013; Zhou and Hu, 2013; Alvira, 2014; Gupta and Sharma, 2014; Laprairie et al., 2014; Snow et al., 2014; Tu et al., 2014; Uekawa et al., 2014; Xiao et al., 2014; Zhou and Zhou, 2014; Zhao et al., 2015), NRF2 (van Muiswinkel and Kuiperij, 2005; Kumar et al., 2012; Sandberg et al., 2013; Arnold et al., 2014; Ding et al., 2014; Chen et al., 2015b; Djordjevic et al., 2015; Zhao et al., 2015), and Notch (John et al., 2002; Minter et al., 2005; Arumugam et al., 2006; Ban et al., 2006; Nagarsheth et al., 2006; Elyaman et al., 2007; Jurynczyk et al., 2008; Yuan and Yu, 2010; Tsugane et al., 2012), as well as between epigenetic phenomena and NF-κB (Arzate-Mejía et al., 2011; Sarnico et al., 2012; Narayan et al., 2015), NRF2 (Martinez et al., 2009; Arzate-Mejía et al., 2011; Gao et al., 2015), and Notch (Arzate-Mejía et al., 2011; Cama et al., 2013; Sun et al., 2014; Schwanbeck, 2015).

To add to the complexity, epigenetic phenomena influence the balance between inflammatory and anti-inflammatory capabilities (Correa et al., 2011; Sarnico et al., 2012; Aithal and Rajeswari, 2013; Cai et al., 2013; Su et al., 2013). As might be expected, systems biologists are paying attention to epigenetic phenomena (Sookoian et al., 2013; Dekker, 2014; Maze et al., 2014; Schadt et al., 2014; Klin et al., 2015; Thakur et al., 2015). Preconditioning/tolerance, the diminished damage that follows a damaging exposure when it is preceded by a sub-injurious exposure (Hagberg et al., 2004), has also attracted the attention of systems biologists (Voit, 2009; Jusko, 2013; León et al., 2013; Gong et al., 2014).

## Part 3. Biomarkers of Exposures and Processes Leading to Brain Damage

Most of the biomarkers of injury to the immature brain are proteins measured in peripheral blood (including, S100B, activin A, adrenomedullin, neuron-specific enolase, oxidative stress markers, glial fibrillary acidic protein, and hemeoxygenase-1) (Douglas-Escobar and Weiss, 2012; Serpero et al., 2013). They give some information about the networks that might have been injured.

Biomarkers of the newborn’s response to potentially adverse exposures, include indicators of systemic inflammation (Leviton et al., 2012; Machado et al., 2014) and other (usually inflammatory) processes associated with injury to the immature brain (Dammann and O’shea, 2008; Malaeb and Dammann, 2009). Markers associated with oxidative stress, including advanced oxidation protein products and total hydroperoxides, malondialdehyde, ascorbate, allantoin (the oxidation product of uric acid) and carbonyl proteins, as well indicators of antioxidant capacity, (vitamins A, E and C) (Negi et al., 2012; Perrone et al., 2012) have the potential to add to our knowledge of the relationship between oxidative stress and encephalopathy of prematurity (Kakita et al., 2009).

In addition, new imaging techniques appear to be increasingly available to identify early structural changes in the brain, (Melbourne et al., 2013; Counsell et al., 2014) just as amplitude EEG is becoming used more widely to assess the maturation and well being of the very preterm newborn (Sohn et al., 2013; Welch et al., 2013; Natalucci et al., 2014). In light of such information about putative-damaging exposures/processes and brain function and structure of very preterm newborns, systems biologists should be able model phenomena related to brain damage in immature humans.

## Part 4. The Future

As those who study systems biology and systems medicine have concentrated on finding a small number of nodes that might serve as targets for therapies and prophylaxis, epidemiologists have concentrated on identifying the exposures that lead to activation of these nodes. For example, in light of our own work, which identified “prolonged” ventilation as a source of postnatal systemic inflammation (Bose et al., 2013), we encourage those responsible for the well-being of very preterm newborns to find and use ventilation strategies that minimize barotrauma and tracheitis (Vendettuoli et al., 2014).

We also identified intermittent and/or sustained systemic inflammation as an antecedent of perinatal brain damage in very preterm newborns (Leviton et al., 2011a,b; O’Shea et al., 2012). With the involvement of elevated concentrations of cytokines, chemokines, adhesion molecules, matrix metalloproteinases, and inflammation-associated growth factors, a therapy that alters the equilibrium at a single, or even a small number of nodes seems incapable of reducing the damage associated with inflammation, let alone contributing to inflammation resolution, and initiating and promoting brain repair. Although inhibitors of a single pro-inflammatory cytokine have diminished the severity of rheumatoid arthritis and inflammatory bowel disease, the most effective appear to have broad effects (Macdonald, 2010). This recognition that a single, highly-specific drug is unlikely to be especially effective has prompted the use of a “cocktail” of multiple drugs targeting multiple inflammatory signaling pathways (Kwon et al., 2013) or a broad spectrum single drug (Belur et al., 2013; Sánchez-Aguilar et al., 2013). Nevertheless, a single, narrow-spectrum inhibitor/modulator might prove effective (Girard et al., 2012; Kight and McCarthy, 2014; Chen et al., 2015a; Zhang et al., 2015). How relevant these efforts are to minimizing brain damage in preterm newborns remains to be seen.

We agree that the best approach is to minimize exposure to brain-damaging insults. Second best is to utilize drugs that target the nodes most likely to be involved (Deboy et al., 2006; Saliba et al., 2007).

Thus, the more information about interrelationships systems biologists and others can provide, the brighter the future for reducing disease burdens on society.

We had four goals in preparing this perspective. First, to show how the antecedents of brain damage can be linked to molecular perturbations. Second, to show how complex and interrelated are the molecular changes that follow exposures that precede the onset of brain damage. Third, to show how a systems approach might expand our understanding, and thereby lead to therapeutic options for encephalopathy of prematurity. Fourth, to call attention to the benefits systems biologists might receive by their study of disturbances to normal brain development, which might provide genetic, proteomic, metabolomic, epigenomic, and microbiomic data needed for models of disease in humans (Nicholson, 2006; Barabási et al., 2011; Espinosa-Jeffrey et al., 2013; Li, 2013; Wolkenhauer, 2013; Wolkenhauer et al., 2013).

## Conflict of Interest Statement

The authors declare that the research was conducted in the absence of any commercial or financial relationships that could be construed as a potential conflict of interest.
